# Different brain profiles in children with prenatal alcohol exposure with or without early adverse exposures

**DOI:** 10.1002/hbm.25130

**Published:** 2020-07-13

**Authors:** Quinn R. Andre, Carly A. McMorris, Preeti Kar, Chantel Ritter, W. Ben Gibbard, Christina Tortorelli, Catherine Lebel

**Affiliations:** ^1^ Medical Science University of Calgary Calgary Alberta Canada; ^2^ Alberta Children’s Hospital Research Institute Calgary Alberta Canada; ^3^ Hotchkiss Brain Institute Calgary Alberta Canada; ^4^ School & Applied Child Psychology University of Calgary Calgary Alberta Canada; ^5^ Department of Pediatrics University of Calgary Calgary Alberta Canada; ^6^ Department of Child Studies and Social Work Mount Royal University Calgary Alberta Canada; ^7^ Department of Radiology University of Calgary Calgary Alberta Canada

**Keywords:** DTI, early adversity, FASD, maltreatment, mental health, MRI, prenatal alcohol exposure

## Abstract

Prenatal alcohol exposure (PAE) can alter brain development and impact mental health outcomes, and often occurs in conjunction with postnatal adversity (e.g., maltreatment). However, it is unclear how postnatal adverse exposures may moderate mental health and brain outcomes in children with PAE. T1‐weighted and diffusion magnetic resonance imaging were obtained from 66 participants aged 7–16 years. Twenty‐one participants had PAE and adverse postnatal exposures (PAE+), 12 had PAE without adverse postnatal exposures (PAE−), and 33 were age‐ and gender‐matched controls unexposed to either prenatal alcohol or postnatal adversity. Internalizing and externalizing mental health symptoms were assessed using the Behavioral Assessment System for Children II, Parent‐Rating Scale. ANCOVAs were used to compare mental health symptoms, limbic and prefrontal cortical volumes, and diffusion parameters of cortico‐limbic white matter tracts between groups, and to assess brain‐mental health relationships. Both PAE groups had worse externalizing behavior (higher scores) than controls. The PAE− group had lower fractional anisotropy (FA) in the bilateral cingulum and left uncinate fasciculus, and smaller volumes in the left anterior cingulate cortex than controls and the PAE+ group. The PAE− group also had higher mean diffusivity (MD) in the left uncinate than the PAE+ group, and smaller right anterior cingulate and superior frontal gyrus volumes than controls. These findings show different brain structure and mental health symptom profiles in children with PAE with and without postnatal adversity, highlighting the need to consider adverse postnatal exposures in individuals with PAE.

## Graphical abstract

We compared mental health symptoms, brain volumes, and diffusion parameters in 21 participants with PAE and adverse postnatal exposures (PAE+), 12 with PAE without adverse postnatal exposures (PAE−), and 33 age‐ and gender‐matched unexposed controls. Both PAE groups had worse externalizing behaviour than controls. The PAE− group had lower fractional anisotropy (FA), higher mean diffusivity, and smaller volumes in several areas compared to controls and the PAE+ group. These findings show different brain structure and mental health profiles in children with PAE with and without postnatal adversity, highlighting the need to consider adverse postnatal exposures in individuals with PAE.

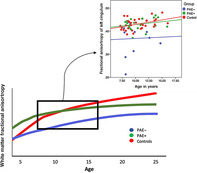

## 1 INTRODUCTION

Alcohol can cross the placenta and blood–brain barrier, directly affecting fetal development and causing long‐term changes in brain and behavior (Uban et al., 2010). Prenatal alcohol exposure (PAE) has been linked with widespread brain abnormalities including reduced brain volume, altered cortical thickness, and altered white matter connectivity (Lebel, Roussotte, & Sowell, 2011; Wozniak & Muetzel, 2011). Fetal alcohol spectrum disorder (FASD), the neurodevelopmental disorder associated with PAE, is the most common cause of preventable developmental disabilities in children (Cook et al., 2016). PAE is also strongly linked with mental health problems; the vast majority of individuals with FASD have at least one mental health disorder (Astley, 2010; Pei, Denys, Hughes, & Rasmussen, 2011). For example, attention deficit hyperactivity disorder (ADHD) occurs in approximately 50% of people with FASD, 10 times the prevalence in the general population (Weyrauch, Schwartz, Hart, Klug, & Burd, 2017). Similarly, anxiety and depression occur in individuals with FASD at a rate of 11 and 4 times the prevalence in the general population, respectively (Weyrauch et al., 2017).

Children with PAE are more likely than the general population to experience adverse postnatal exposures such as neglect, poverty, caregiver transitions, abuse (verbal, physical, and sexual), and witnessing violence or chronic substance use (Astley, 2010; Lebel et al., 2019). These postnatal experiences can negatively affect brain development (Bick et al., 2015; Hart & Rubia, 2012) and have been associated with increased internalizing (inwardly directed negative behaviors such as anxiety and depression) and externalizing‐based problems and disorders (outwardly directed negative behaviors such as aggression and hyperactivity) (Felitti et al., 1998; Flaherty et al., 2013; Henry, Sloane, & Black‐Pond, 2007; Kerker et al., 2015). The vast majority of previous studies of PAE do not address co‐occurring postnatal exposures (Fryer et al., 2009; Nardelli, Lebel, Rasmussen, Andrew, & Beaulieu, 2011), though a few studies have demonstrated that children and adolescents with FASD and postnatal trauma have more severe externalizing behaviors than children and adolescents with postnatal trauma and no PAE (Henry et al., 2007; Hyter, 2012; Price et al., 2017). Another study showed that children with FASD who lived with their biological parents had worse behavior problems and worse developmental delays than children with FASD who were adopted at birth (Koponen, Kalland, & Autti‐Rämö, 2009; Koponen, Kalland, Autti‐Rämö, Laamanen, & Suominen, 2013).

PAE has been linked with volume reductions in the frontal lobe (Astley et al., 2009), including the middle frontal gyri in the prefrontal cortex (PFC) (Eckstrand et al., 2012), as well as deep gray matter structures such as the hippocampus and amygdala (Astley et al., 2009; Bookstein, Sampson, Connor, & Streissguth, 2002; Nardelli et al., 2011; Sowell et al., 2001; Wozniak et al., 2013). White matter also shows widespread effects of PAE, including lower fractional anisotropy (FA), and/or higher mean diffusivity (MD) in limbic tracts, such as the uncinate fasciculus and cingulum (Fryer et al., 2009; Lebel, Rasmussen, et al., 2008a). Postnatal adversity is associated with brain changes in similar areas, specifically smaller PFC and hippocampal volumes, increased amygdala volume (Andersen et al., 2008; Hanson et al., 2010; Pechtel, Lyons‐Ruth, Anderson, & Teicher, 2014), and decreased FA and increased MD in the cingulum and fornix (Bick et al., 2015; Choi, Jeong, Rohan, Polcari, & Teicher, 2009; Dufford & Kim, 2017). While PAE and postnatal adversity often co‐occur, no previous neuroimaging studies of PAE have also considered postnatal exposures, so it remains unclear whether postnatal exposures moderate the effects of PAE on brain structure.

The goal of this study was to determine how PAE in the presence or absence of postnatal adverse exposures is associated with brain structure and mental health symptoms in children. Based on their role in mental health symptoms (Ahmed, Bittencourt‐Hewitt, & Sebastian, 2015; Mincic, 2015), as well as alterations in individuals with PAE (Coles et al., 2011; Lebel, Rasmussen, et al., 2008a; Nardelli et al., 2011) and those with postnatal adversity (Bick & Nelson, 2016; Dufford & Kim, 2017), we examined prefrontal (anterior cingulate cortex, superior frontal, and middle frontal gyri) and limbic (amygdala and hippocampus) structures, as well as their corresponding white matter connections (cingulum, fornix, and uncinate fasciculus). We hypothesized that children and adolescents with PAE and adverse postnatal exposures would have lower volumes and FA and higher MD in these structures, as well as increased mental health symptoms compared to children with PAE and no postnatal adversity, and unexposed controls. That is to say, we predicted there would be cumulative effects of prenatal and postnatal exposures in these regions, such that the combined exposures would result in similar but stronger effects in comparison to those with prenatal alcohol exposure alone. We further predicted that more neural changes would be associated with worse (increased) mental health symptoms.

## 2 METHODS

### 2.1 Participants

Thirty‐three children and adolescents with PAE aged 7–16 years and 33 age‐ and gender‐matched unexposed controls were recruited through posters, social media, newsletters, word of mouth, and (for participants with PAE) through the Cumulative Risk Diagnostic Clinic located in Calgary, Alberta. Gender was determined through parent report. Controls had no prior diagnoses of mental health disorders or neurodevelopmental disorders. Most participants with PAE had prior diagnoses of ADHD (*n* = 8 with PAE−, 17 with PAE+), anxiety (*n* = 1 with PAE−, 2 with PAE+), learning disability (*n* = 2 with PAE−, 4 with PAE+), oppositional defiant disorder (*n* = 2 with PAE−, 3 with PAE+), Tourette syndrome (n = 1 with PAE−, 1 with PAE+), attachment disorder (*n* = 0 with PAE−, 2 with PAE+), and/or obsessive–compulsive disorder (*n* = 1 with PAE−, 0 with PAE+); 12 participants had more than one diagnosis (excluding a diagnosis of FASD). Two participants with PAE alone, and 1 participant with PAE and postnatal adversity had no other diagnoses. All participants had no MRI contraindications. Written informed consent and assent were obtained from parents/guardians and participants, respectively. This study was approved by the University of Calgary Conjoint Health Research Ethics Board (REB 17‐0663). Table 1 outlines participant demographics of each group.

**TABLE 1:** Demographics for the prenatal alcohol exposed group without adverse postnatal exposures (PAE−), the prenatal alcohol exposed group with adverse postnatal exposures (PAE+), and unexposed controls

	PAE−	PAE+	Controls
Gender	7 M/5F	14 M/7F	21 M/12F
Age	9.8 ± 2.2 years	10.7 ± 2.3 years	10.4 ± 2.4 years
Maternal education	14.9 ± 2.7 years	14.6 ± 2.2 years	15.5 ± 1.9 years
Household income	$75,000–99,999	$100,000–124,999	$100,000–124,999

*Note:* Maternal education and household income for the PAE groups pertain to their guardians in their placements at the time of the study. No significant group differences were found for these variables. See Section 2.3 for further details about groupings.

### 2.2 Behavioral measures

Internalizing and externalizing mental health symptoms were measured using the Behavioral Assessment System for Children, Second Edition—Parent Rating Scale (BASC‐2‐PRS; Reynolds, Kamphaus, & Vannest, 2011). The BASC‐2 computes T‐scores for internalizing behaviors and externalizing behaviors. It is used for both clinical and research purposes, provides high sensitivity to DSM III and IV diagnoses, convergent and discriminant validity, high internal consistency and temporal stability (Baxter & Rattan, 2004; Doyle, Ostrander, Skare, Crosby, & August, 1997; Gladman & Lancaster, 2003; Jarratt, Riccio, & Siekierski, 2005; Vaughn, Riccio, Hynd, & Hall, 2010). It is a continuous scale with higher scores indicating more prominent displays of the emotion or behavior. A T‐score of 50 is the mean for the general population. In clinical settings, cut‐offs can be utilized to aid in diagnostic processes. A T‐score ≥60 is considered at‐risk, while a score ≥70 indicates clinically significant levels of maladaptive behaviors and suggests that children may meet diagnostic criteria for an emotional or behavioral disorder. Two PAE participants were missing BASC‐2 scores due to incomplete forms.

### 2.3 Assessment of exposures

All non‐control participants had PAE confirmed by maternal report, direct observation by close friends or family of the mother drinking alcohol while pregnant, and/or positive maternal blood or urine test in pregnancy. Postnatal adversity was defined as exposures that put a child at risk for altered developmental outcomes and included abuse or witnessing abuse; neglect; insecurity of food, housing, or income; and caregiver changes. Information about postnatal exposures was obtained through assessments, questionnaires, and interviews from biological and foster/adoptive families, and documentation from child welfare records, physicians, police reports, and social workers. Each participant's information was evaluated by a committee to characterize their pre‐ and postnatal adversity. Absence of postnatal adversity meant a participant had no reports of adverse exposures and no caregiver changes from birth. Due to a relatively small sample, we assessed presence or absence of all postnatal adversity in the children and youth with PAE rather than subdividing postnatal adversity into types of exposure (Lebel et al., 2019). Twelve participants with PAE did not have any postnatal adversity (PAE−), and 21 participants with PAE had some form of postnatal adversity (PAE+). In this sample, all participants with an absence of adverse postnatal exposures were adopted or in kinship care placements from birth. One PAE+ participant still lived with a biological parent. The age at which children were placed in a stable home environment (i.e., when they entered their current, permanent home, typically via adoption) was obtained from child welfare records and interviews with caregivers for use in a follow‐up analysis.

### 2.4 Brain imaging

Participants were scanned on a 3T GE MR750w MRI scanner at the Alberta Children's Hospital using a 32‐channel head coil. T1‐weighted anatomical imaging was acquired using a 3D FSPGR sequence with TI = 600 ms, TR = 8.2 ms, and TE = 3.2 ms, 0.8 mm isotropic resolution, and a total scan time of 5:38 min. Freesurfer v5.3 was used for processing, editing, and segmenting the T1‐weighted anatomical images (Fischl, 2012). The automated recon‐all pipeline (Dale, Fischl, & Sereno, 1999) was applied to register the individual brains to a template brain and complete brain extraction, image registration, intensity correction, segmentation/parcellation, and volume calculation. All segmentations were manually checked to ensure proper delineation of the outer pial and white matter border, and manual editing (adding control points to denote white matter voxels) was performed when necessary. Volumes of the amygdala, hippocampus, anterior cingulate cortex, superior frontal gyrus, and middle frontal gyrus were extracted. Two participants' PFC volumes were removed (bilateral anterior cingulate, middle frontal, and superior frontal gyri) due to poor segmentation.

**FIGURE 1:**
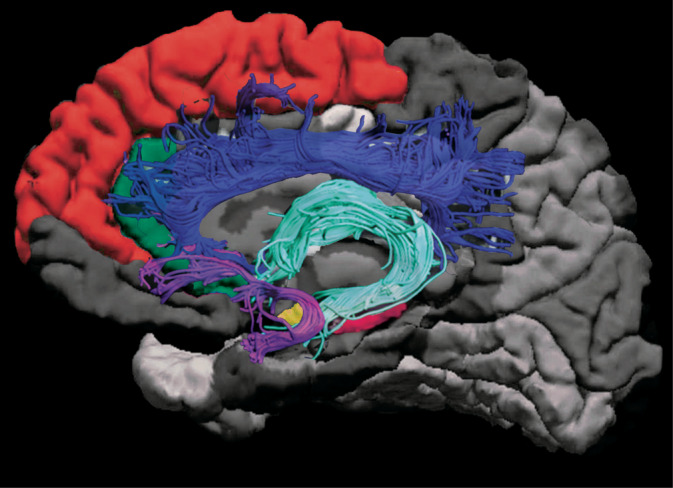
Diagram of measured brain structures. For anatomical volumes, the amygdala (yellow), hippocampus (pink), anterior cingulate cortex (green), superior frontal gyrus (red), and the middle frontal gyrus (not shown), were assessed. For DTI tractography, the uncinate fasciculus (magenta), fornix (cyan), and the cingulum (blue), were assessed

Diffusion tensor imaging (DTI) was acquired with a spin echo echo planar imaging (EPI) sequence using 30 gradient‐encoding diffusion directions at *b* = 900 s/mm^2^ and five images at *b* = 0 s/mm^2^, TR = 12 s, TE = 88 ms, 2.2 mm isotropic resolution, and a total scan time of 7:12 min:sec. ExploreDTI (Leemans, Jeurissen, Sijbers, & Jones, 2009) was used for diffusion‐weighted image processing, including correction for signal drift (Vos et al., 2017), Gibb's ringing (Perrone et al., 2015), subject motion, eddy current distortion (Leemans & Jones, 2009), and EPI distortion (Irfanoglu, Walker, Sarlls, Marenco, & Pierpaoli, 2012). Constrained spherical deconvolution (Jeurissen, Leemans, Jones, Tournier, & Sijbers, 2011) was used to compute a whole brain tractogram. Semi‐automated tractography (Lebel, Rasmussen, et al., 2008a) was performed to extract the cingulum, fornix, and uncinate fasciculus using regions of interest based on a priori knowledge of tract location (Abdul‐Rahman, Qiu, & Sim, 2011; Larroza, Moratal, D'ocon Alcaniz, & Arana, 2014; Lebel, Walker, et al., 2008b; Plaisier et al., 2014; Wakana, Jiang, Nagae‐Poetscher, van Zijl, & Mori, 2004). Manual checks of each tract were done to ensure quality of the segmentations and edits were performed when necessary. Average FA and MD were calculated for each tract for each participant, separately for the left and right hemispheres. Figure 1 shows the volumes and tracts assessed.

### 2.5 Statistical analysis

IBM SPSS Statistics, Version 24 (IBM Corp., Armonk, NY) was used for all statistical analyses. Mental health symptoms, T1‐weighted volumes, and DTI measures (FA and MD) were tested with separate ANCOVAs, with group and gender as factors and age as a covariate. Interaction terms were included but removed from the model if not significant. False discovery rate (FDR) was used to account for 2 tests for mental health symptoms, 12 tests using DTI measures (3 tracts*2 hemispheres*2 measures), and 10 tests using T1 measures (5 regions*2 hemispheres). Scheffe post hoc tests were used to determine significant between group differences. ANCOVAs were then used to test relationships between brain structure and mental health symptoms with group interactions. FDR corrections for multiple comparisons were used. Age and behavioral measure (internalizing or externalizing) were included as covariates, and gender was included as a factor.

Follow‐up analysis used ANCOVAs (mental health symptoms, T1‐weighted volumes, DTI measures) to test relationships between mental health and brain measures and age at stable placement for PAE participants who experienced caregiver changes (i.e., those with age at stable placement >0) with age as a covariate and gender as a factor. Results were corrected for multiple comparisons with FDR.

## 3 RESULTS

### 3.1 Postnatal exposures

Of the children and adolescents with PAE, 64% had adverse postnatal exposures, of which 42% experienced abuse, 47% experienced neglect (24% experienced both), 38% were witness to domestic violence, 25% were witness to substance abuse, and all but one child had caregiver changes; many children had multiple exposures.

### 3.2 Internalizing and externalizing mental health symptoms

**FIGURE 2:**
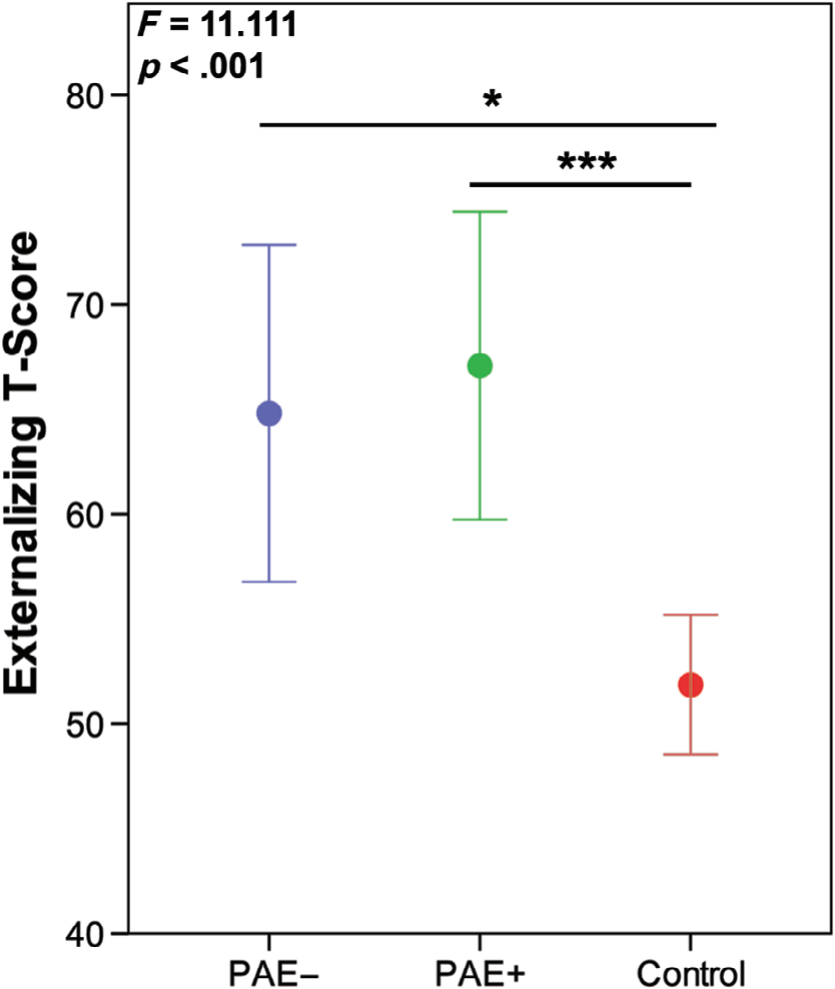
Children and adolescents with prenatal alcohol exposure and no adverse postnatal exposures (PAE−) (blue) and those with PAE and adverse postnatal exposures (PAE+) (green) had higher externalizing scores than unexposed controls (red). *Indicates significant between group differences *p* ≤ .05, and *** indicates between group differences *p* ≤ .001. *F* and *p*‐values indicate overall group differences on the ANCOVA

The ANCOVA for externalizing mental health symptoms had significant between subject effects for group (*F* = 11.111, *p* < .001), with the PAE alone (PAE−) and PAE with postnatal adversity (PAE+) groups having higher externalizing scores than controls (*p* = .016, *p* < .001, respectively; Figure 2). Table 2 outlines behavioral means, and prevalence of at‐risk and clinically significant scores for each group.

**TABLE 2:** Mean *T*‐scores and prevalence of at risk and clinically significant levels of internalizing and externalizing scores for the prenatal alcohol exposed group without adverse postnatal exposure (PAE−), the prenatal alcohol exposed group with adverse postnatal exposures (PAE+), and unexposed controls

		PAE−(*n* = 11)	PAE+(*n* = 20)	Controls (*n* = 33)	ANCOVA
*Internalizing scores*	*Mean score*	57 ± 19	57 ± 17	49 ± 10	*F* = 2.345 *p* = .105
	At risk (≥60)	9% (1/11)	10% (2/20)	6% (2/33)	
	Clinically significant (≥70)	27% (3/11)	25% (5/20)	6% (2/33)	
	Total	36%	35%	12%	
*Externalizing scores*	*Mean score*	65 ± 11^a^	67 ± 16^a^	52 ± 10	*F* = 11.111 *p* < .001
	At risk (≥60)	45% (5/11)	50% (10/20)	12% (4/33)	
	Clinically significant (≥70)	27% (3/11)	30% (6/20)	6% (2/33)	
	Total	72%	80%	18%	

*Note:* The PAE− and PAE+ groups each had one incomplete behavioral assessment.

^a^ Mean scores significantly higher than controls.

### 3.3 White matter

**FIGURE 3:**
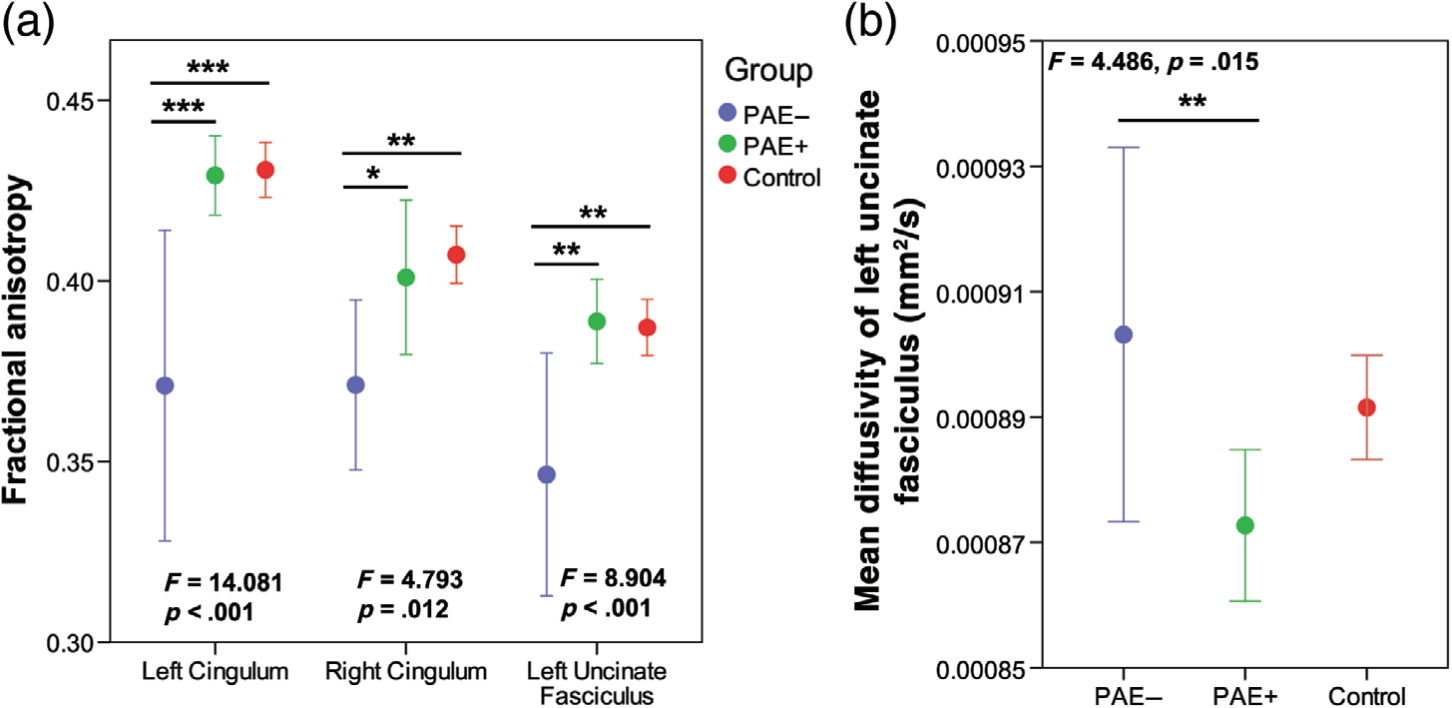
(a) Children and adolescents with prenatal alcohol exposure and no adverse postnatal exposures (PAE−) (blue) had significantly lower FA than those with PAE and adverse postnatal exposures (PAE+) (green) and unexposed controls (red) in the cingulum and left uncinate. (b) The PAE+ group had significantly lower MD than the PAE− group in the left uncinate fasciculus. * Indicates significant between group differences *p* ≤ .05, ** indicates between group differences *p* ≤ .01, and *** indicates between group differences *p* ≤ .001. *F* and *p*‐values indicate overall group differences

The ANCOVA for white matter measures revealed a significant between subject effects for group for FA of the bilateral cingulum (Left: *F* = 14.081, *p* < .001; Right: *F* = 4.793, *p* = .012) and left uncinate fasciculus (*F* = 8.904, *p* < .001), and MD of the left uncinate fasciculus (*F* = 4.486, *p* = .015). Post hoc tests revealed that the PAE− group had lower FA than controls and the PAE+ group in the left cingulum (both *p* < .001), right cingulum (controls: *p* = .009; PAE+: *p* = .034) and left uncinate fasciculus (both *p* = .001; Figure 3a). The PAE− group had higher MD than the PAE+ group in the left uncinate (*p* = .01; Figure 3b).

### 3.4 Brain volumes

**FIGURE 4:**
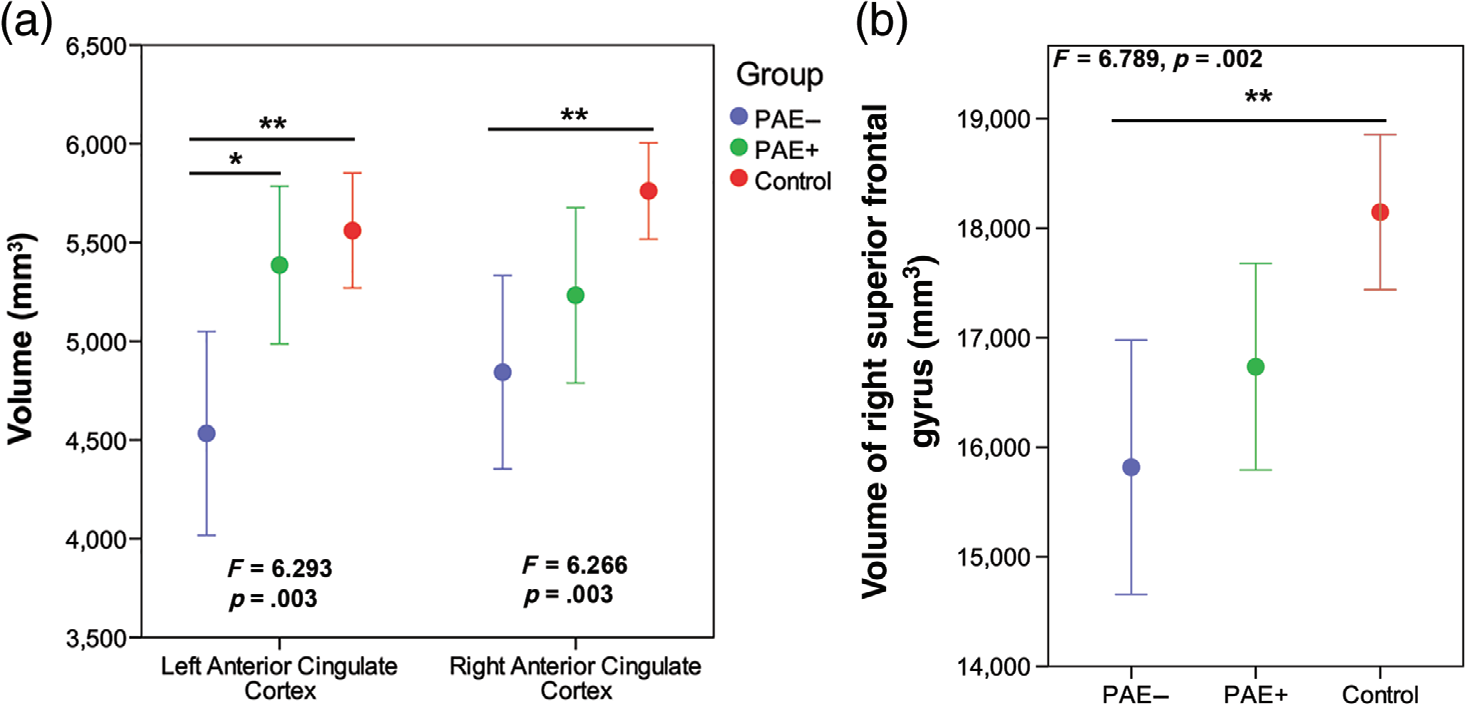
(a) Children and adolescents with prenatal alcohol exposure and no adverse postnatal exposures (PAE−) (blue) had significantly lower volume than unexposed controls (red) in the left and right anterior cingulate cortex and lower volume than those with PAE and adverse postnatal adversities (PAE+) (green) in the left anterior cingulate cortex. (b) The PAE− had lower volume than controls in the right superior frontal gyrus. * Indicates significant between group differences *p* ≤ .05 and ** indicates between group differences *p* ≤ .01. *F* and *p*‐values indicate overall group differences

The ANCOVA for brain volumes revealed a between‐subjects effect of group in the bilateral anterior cingulate cortex (Left: *F* = 6.293, *p* = .003; Right: *F* = 6.266, *p* = .003), and right superior frontal gyrus volume (*F* = 6.789, *p* = .002). Post hoc tests revealed that the PAE− group had smaller volumes than controls in the bilateral anterior cingulate cortex (Left: *p* = .005; Right: *p* = .007; Figure 4a) and right superior frontal gyrus (*p* = .005; Figure 4b). The PAE− group had smaller volumes than PAE+ participants in the left anterior cingulate cortex (*p* = .033).

Generally, the results in the PAE− group supported the hypothesized neural relationships when compared to controls (i.e., they had lower FA and smaller volumes). The PAE+ group results were more surprising, since they did not show hypothesized relationships compared to controls or the PAE− group in most cases. The PAE+ group showed similar FA values to controls, and higher FA and volumes and lower MD values than the PAE− group. Overall, this sample achieved 75–99% power to detect the medium to large effect sizes (*f* = 0.37–0.68) observed for the neural results. Similar effect sizes and results have been reported in previous PAE research (Lebel, Rasmussen, et al., 2008a; Sowell et al., 2008; Wozniak et al., 2006; Wozniak & Muetzel, 2011).

### 3.5 Relationships between brain and mental health measures

ANCOVAs did not show any significant brain‐behavior interaction for internalizing or externalizing behavior.

### 3.6 Age at stable placement

ANCOVA for age at stable placement for PAE participants who experienced caregiver changes revealed a between subjects effect for age at stage placement for FA of the left cingulum (*F* = 7.665, *p* = .014), but this result did not survive FDR correction.

## 4 DISCUSSION

Here, we show for the first time that children and youth with PAE have different brain structure depending on the presence or absence of adverse postnatal exposures. Children and adolescents with PAE, regardless of postnatal adversity, showed more mental health symptoms than unexposed controls. However, the group with PAE and no adverse postnatal exposures (PAE−) showed more widespread structural brain differences from controls than the group with PAE and adverse postnatal exposures (PAE+), suggesting that prenatal and postnatal exposures may interact to influence brain development in different ways.

Both PAE groups had more externalizing symptoms than controls. 72 and 80% of the PAE− and PAE+ groups, respectively, had at risk or clinical levels of externalizing symptoms, compared to only 18% of unexposed controls. Symptoms were not significantly different between the PAE groups with and without postnatal exposure. Previous research shows elevated risk of mental health disorders in children with PAE (Pei et al., 2011), or with postnatal trauma (Norman et al., 2012), though the majority of previous work did not consider other exposures (i.e., postnatal exposure studies do not ask about PAE, and PAE studies do not report postnatal adversities). One study showed that children with FASD and postnatal trauma had worse externalizing symptoms than children with postnatal trauma alone (Henry et al., 2007). Thus, PAE itself appears to be important in the mental health difficulties of children and youth, and additional postnatal trauma may exacerbate these issues.

Structural brain differences were prevalent in the PAE− group, which had lower FA in the cingulum and uncinate fasciculus compared to controls. This is consistent with earlier studies showing lower FA in the cingulum and uncinate fasciculus in children and youth with FASD (Fryer et al., 2009; Lebel, Rasmussen, et al., 2008a). Prefrontal cortical regions, including the anterior cingulate cortex and superior frontal gyrus, also had lower volumes in the PAE− group compared to controls, consistent with previous findings of widespread smaller gray matter volumes in PAE (Coles et al., 2011; Lebel et al., 2011; Lebel, Rasmussen, et al., 2008a).

**FIGURE 5:**
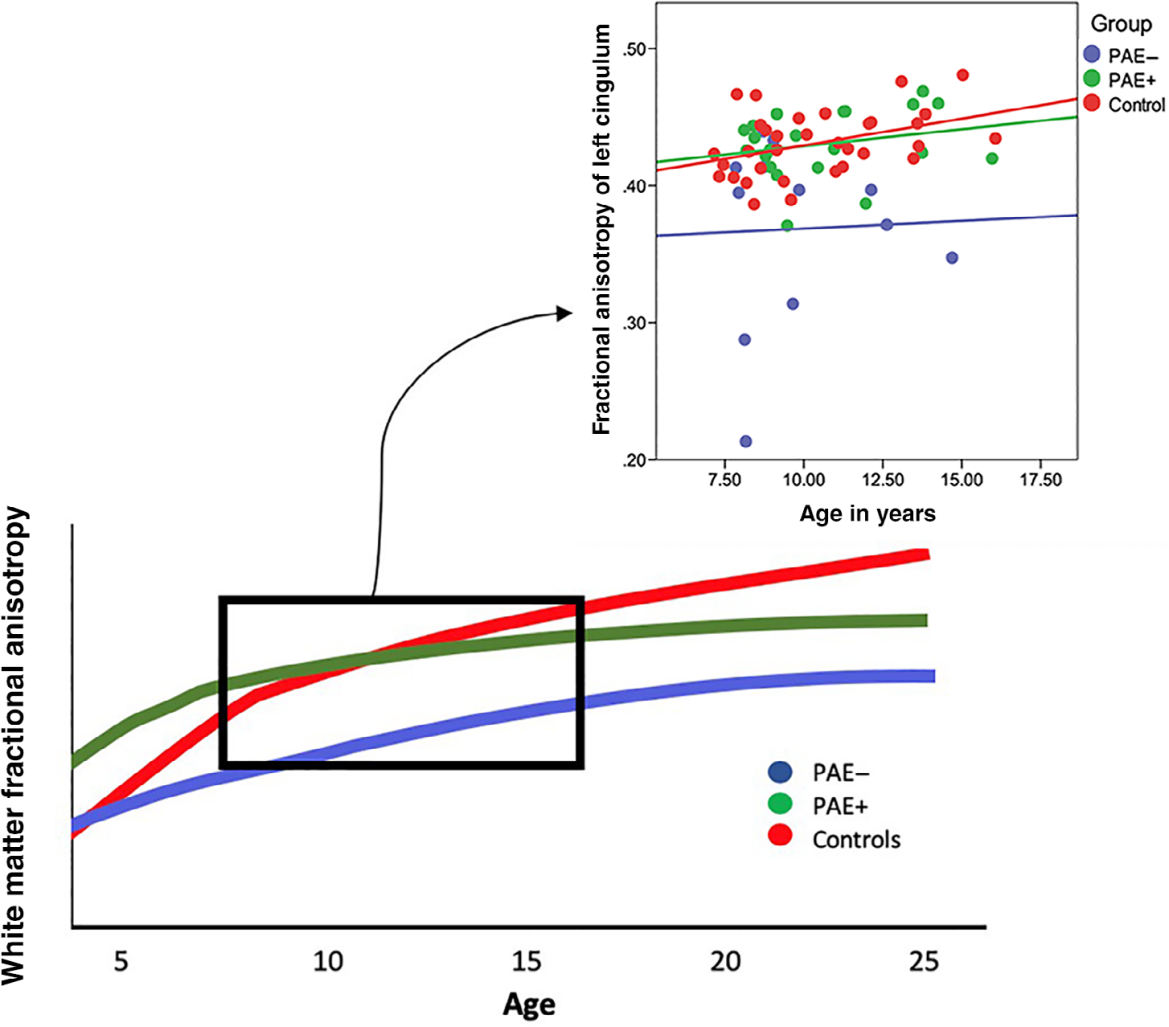
Visual depiction of altered structural brain trajectories hypothesized for white matter fractional anisotropy (FA) in individuals with PAE with (PAE+; green) or without (PAE−; blue) postnatal adversity compared to controls (red) based on FA of the left cingulum and age relationship. The inset scatterplot shows data from this study, which provides some support for this hypothesis, though does not have adequate power to assess age‐group interactions

Surprisingly, the PAE+ group showed similar brain structure to controls, suggesting that postnatal adversity in conjunction with PAE results in different brain outcomes than PAE alone. The PAE+ group had higher FA in the cingulum and uncinate fasciculus, lower MD in the left uncinate, and larger volumes of the left anterior cingulate cortex than the PAE− group. Some diffusion imaging studies have shown higher MD after institutional neglect (Bick et al., 2015), and lower FA associated with low family income (Dufford & Kim, 2017) and verbal abuse (Choi et al., 2009), though no information about PAE was reported. Other studies have shown higher FA in the cingulum, uncinate fasciculus, anterior thalamic radiation, and forceps minor in adolescents and adults after childhood neglect and adversity (Hanson et al., 2013; Ugwu, Amico, Carballedo, Fagan, & Frodl, 2015), though again no information on PAE was reported. Lower MD in parts of the corpus callosum and anterior corona radiata have been reported in children of mothers with increased postpartum depressive symptoms (Lebel et al., 2016). Similarly, previously reported gray matter alterations include smaller superior frontal and dorsolateral PFC volumes, but larger cingulate volumes in children who had been abused (Hanson et al., 2010), as well as larger amygdala volumes in both humans and rodents after maternal absence (Callaghan, Sullivan, Howell, & Tottenham,^ 2014^). It may be that PAE leads to lower FA, higher MD, and smaller volumes, but that some adverse postnatal exposures accelerate brain development, resulting in little‐to‐no measurable difference between the PAE+ group and controls during late childhood or early adolescence. In this case, accelerated development would be considered sub‐optimal, and is likely to be accompanied by an earlier developmental plateau (i.e., development stops at a younger age than in typically‐developing children). In fact, premature brain development often results in later underdevelopment, such that plateaus are reached sooner and at ultimately lower levels (e.g., lower FA and smaller volumes; Courchesne et al., 2007; Deoni et al., 2016; Shaw et al., 2006). Limbic white matter tracts and prefrontal brain regions show very protracted development (Gogtay et al., 2004; Lebel, Walker et al., 2008b), and thus differences between children and youth with PAE and postnatal exposures may become more pronounced at older ages. Figure 5 shows hypothesized trajectories of brain development in children with PAE with or without postnatal adversity, along with data from the current study. Although our data is cross‐sectional, it appears to support the idea that unexposed controls may continue developing after individuals with PAE+ have plateaued. Longitudinal studies will be needed to properly detail the trajectories of brain development in children with PAE with or without postnatal exposures.

We did not find significant group interactions in the relationships between brain structure and mental health behavior. This is likely due to small samples and minimal power to detect interaction effects. Previous work has shown relationships between brain structure and mental health symptoms in typically‐developing children and adolescents (Ali, Vandermeer, Sheikh, Joanisse, & Hayden, 2019; Ameis et al., 2014; Andre, Geeraert, & Lebel, 2019; Boes, Tranel, Anderson, & Nopoulos, 2008; Ducharme et al., 2011, 2014; Koolschijn, van IJzendoorn, Bakermans‐Kranenburg, & Crone, 2013; Snyder, Hankin, Sandman, Head, & Davis, 2017; van der Plas, Boes, Wemmie, Tranel, & Nopoulos, 2010; Yap, Whittle, Yücel, & Sheeber, 2008) but our study is the first to address the neural correlates of mental health symptoms in a PAE population. Mental health treatments are less effective in individuals with PAE (Anderson, Mela, & Stewart, 2017), which may be partially explained by different brain structure. Future work with larger samples will be vital to gain a better understanding of the link between brain structure and mental health symptoms across groups, and to determine if there are group differences in the way neural correlates are related to mental health symptoms. Individuals with PAE may require unique developmental and mental health surveillance, treatments, and/or services to address their specific needs while acknowledging the potential impact of their postnatal environments.

A limitation of this study is the use of parent‐rating questionnaires for mental health symptoms, which can differ from child self‐reports and do not necessarily provide a full assessment of a child's behaviors (Barnhill et al., 2000; Kolko & Kazdin, 1993). Future research including child self‐reports will further expand our knowledge of brain‐behavior relationships. Additionally, a matched sample of children and adolescents with neurodevelopmental and/or mental health disorders without PAE would provide insight as to the impact of these conditions on the brain independently, as most (91%) of our PAE sample had other diagnoses. Another limitation was the broad age range of our sample. This allowed us to have a larger sample size and to investigate age‐group interactions as well as age of stable placement, but it also introduced a large range of neurodevelopment. Neural structures and mental health status may be differentially affected at age 7 than at 16. This was mitigated in analyses by controlling for age and including Table S1 results of group‐by‐age interactions. We used extensive documentation and interviews to characterize prenatal and postnatal exposures as comprehensively as possible, although some unknowns still remain. For example, the amount, frequency, and duration of alcohol consumption during pregnancy was not always available. Finally, there is evidence that different types of early adversity (e.g., threat vs. deprivation) impact the brain in different ways (McLaughlin & Sheridan, 2016), however, our sample was too small to separate different types of postnatal adversity. Future research with larger samples will be necessary to further understand these relationships.

In conclusion, children and youth with PAE had more externalizing symptoms than controls regardless of postnatal exposures, while children with PAE and no postnatal exposures had differences in brain structure from controls. These findings suggest that prenatal exposures and postnatal experiences interact with brain development differently. The unique and divergent effects of adverse exposures, pre‐ and postnatal, on mental health symptoms and brain developmental trajectories highlights the need for recognition of multiple exposures in PAE research.

## CONFLICT OF INTEREST

Catherine Lebel's spouse is an employee of General Electric Healthcare. The authors report no other biomedical financial interests or potential conflicts of interest.

## Supporting information


**Table S1** Between‐subject effects for the group‐by‐age interaction for white matter measures and gray matter volumes for the prenatal alcohol exposure alone (PAE−), prenatal alcohol exposure with postnatal adversities (PAE+), and control groups. None of the group‐by‐age interactions were statistically significant.

## Data Availability

Neuroimaging data for the unexposed control group is publicly available here: https://doi.org/10.6084/m9.figshare.6002273.v2 (Lebel, 2018). Data on the PAE groups are available on request from the corresponding author.
