# Supplementary material for: Different brain profiles in children with prenatal alcohol exposure with or without early adverse exposures
Source: Hum Brain Mapp. 2020 Jul 13;41(15):4375–85. doi: 10.1002/hbm.25130 (PMC7502833; doi:10.1002/hbm.25130)
Supplement: Supplementary file 1 — Table S1 Between‐subject effects for the group‐by‐age interaction for white matter measures and gray matter volumes for the prenatal alcohol exposure alone (PAE−), prenatal alcohol exposure with postnatal adversities (PAE+), and control groups. None of the group‐by‐age interactions were statistically significant. [file HBM-41-4375-s001.docx]

**Supplementary Table**

**Supplementary Table 1.** Between-subject effects for the group-by-age interaction for white matter measures and gray matter volumes for the prenatal alcohol exposure alone (PAE-), prenatal alcohol exposure with postnatal adversities (PAE+), and control groups. None of the group-by-age interactions were statistically significant.

| **White Matter Measures** | **F-Values** | **p-Values** | **Gray Matter Volumes** | **F-Values** | **p-Values** |
| --- | --- | --- | --- | --- | --- |
| FA Left Cingulum | 0.196 | 0.823 | Left Anterior Cingulate | 0.578 | 0.564 |
| FA Right Cingulum | 0.755 | 0.474 | Right Anterior Cingulate | 0.752 | 0.476 |
| FA Left Uncinate | 0.368 | 0.693 | Left Middle Frontal | 0.329 | 0.721 |
| FA Right Uncinate | 0.078 | 0.925 | Right Middle Frontal | 0.028 | 0.972 |
| FA Left Fornix | 0.695 | 0.503 | Left Superior Frontal | 2.041 | 0.139 |
| FA Right Fornix | 0.050 | 0.951 | Right Superior Frontal | 0.974 | 0.384 |
| MD Left Cingulum | 0.060 | 0.941 | Left Hippocampus | 0.488 | 0.616 |
| MD Right Cingulum | 0.000 | 1.000 | Right Hippocampus | 1.157 | 0.321 |
| MD Left Uncinate | 1.752 | 0.182 | Left Amygdala | 0.151 | 0.860 |
| MD Right Uncinate | 0.639 | 0.531 | Right Amygdala | 0.408 | 0.667 |
| MD Left Fornix | 1.430 | 0.247 |  |  |  |
| MD Right Fornix | 0.558 | 0.575 |  |  |  |
